# Elucidation of the molecular mechanism of the breakage-fusion-bridge (BFB) cycle using a CRISPR-dCas9 cellular model

**DOI:** 10.1093/nar/gkae747

**Published:** 2024-08-28

**Authors:** Manrose Singh, Kaitlin Raseley, Alexis M Perez, Danny MacKenzie, Settapong T Kosiyatrakul, Sanket Desai, Noelle Batista, Navjot Guru, Katherine K Loomba, Heba Z Abid, Yilin Wang, Lars Udo-Bellner, Randy F Stout, Carl L Schildkraut, Ming Xiao, Dong Zhang

**Affiliations:** Department of Biomedical Sciences, College of Osteopathic Medicine, New York Institute of Technology, Old Westbury, NY 11568, USA; School of Biomedical Engineering, Science and Health System, Drexel University, Philadelphia, PA 19104, USA; Department of Biomedical Sciences, College of Osteopathic Medicine, New York Institute of Technology, Old Westbury, NY 11568, USA; Department of Biomedical Sciences, College of Osteopathic Medicine, New York Institute of Technology, Old Westbury, NY 11568, USA; Department of Cell Biology, Albert Einstein College of Medicine, Bronx, NY 10461, USA; Department of Biomedical Sciences, College of Osteopathic Medicine, New York Institute of Technology, Old Westbury, NY 11568, USA; Department of Biomedical Sciences, College of Osteopathic Medicine, New York Institute of Technology, Old Westbury, NY 11568, USA; Department of Biomedical Sciences, College of Osteopathic Medicine, New York Institute of Technology, Old Westbury, NY 11568, USA; Department of Biomedical Sciences, College of Osteopathic Medicine, New York Institute of Technology, Old Westbury, NY 11568, USA; School of Biomedical Engineering, Science and Health System, Drexel University, Philadelphia, PA 19104, USA; School of Biomedical Engineering, Science and Health System, Drexel University, Philadelphia, PA 19104, USA; Department of Biomedical Sciences, College of Osteopathic Medicine, New York Institute of Technology, Old Westbury, NY 11568, USA; Department of Biomedical Sciences, College of Osteopathic Medicine, New York Institute of Technology, Old Westbury, NY 11568, USA; Department of Cell Biology, Albert Einstein College of Medicine, Bronx, NY 10461, USA; School of Biomedical Engineering, Science and Health System, Drexel University, Philadelphia, PA 19104, USA; Center for Genomic Sciences and Center for Advanced Microbial Processing, Institute of Molecular Medicine and Infectious Disease, Drexel University College of Medicine, Philadelphia, PA, 19102, USA; Department of Biomedical Sciences, College of Osteopathic Medicine, New York Institute of Technology, Old Westbury, NY 11568, USA; Center for Cancer Research, New York Institute of Technology, Old Westbury, NY 11568, USA

## Abstract

Chromosome instability (CIN) is frequently observed in many tumors. The breakage-fusion-bridge (BFB) cycle has been proposed to be one of the main drivers of CIN during tumorigenesis and tumor evolution. However, the detailed mechanism for the individual steps of the BFB cycle warrants further investigation. Here, we demonstrate that a nuclease-dead Cas9 (dCas9) coupled with a telomere-specific single-guide RNA (sgTelo) can be used to model the BFB cycle. First, we show that targeting dCas9 to telomeres using sgTelo impedes DNA replication at telomeres and induces a pronounced increase of replication stress and DNA damage. Using Single-Molecule Telomere Assay via Optical Mapping (SMTA-OM), we investigate the genome-wide features of telomeres in the dCas9/sgTelo cells and observe a dramatic increase of chromosome end fusions, including fusion/ITS+ and fusion/ITS−. Consistently, we also observe an increase in the formation of dicentric chromosomes, anaphase bridges, and intercellular telomeric chromosome bridges (ITCBs). Utilizing the dCas9/sgTelo system, we uncover many interesting molecular and structural features of the ITCB and demonstrate that multiple DNA repair pathways are implicated in the formation of ITCBs. Our studies shed new light on the molecular mechanisms of the BFB cycle, which will advance our understanding of tumorigenesis, tumor evolution, and drug resistance.

## Graphical Abstract

**Graphical Abstract:**
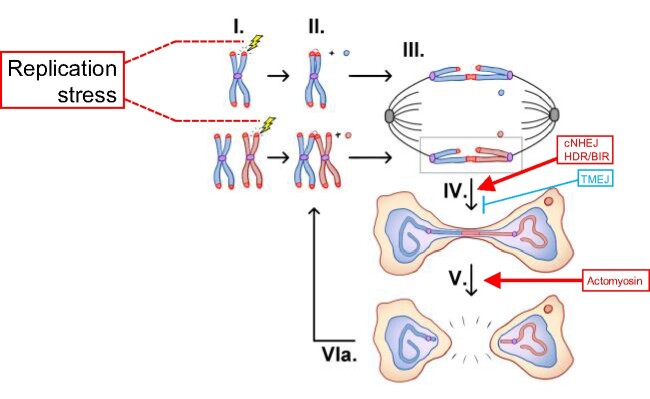

## Introduction

**Figure 1.**
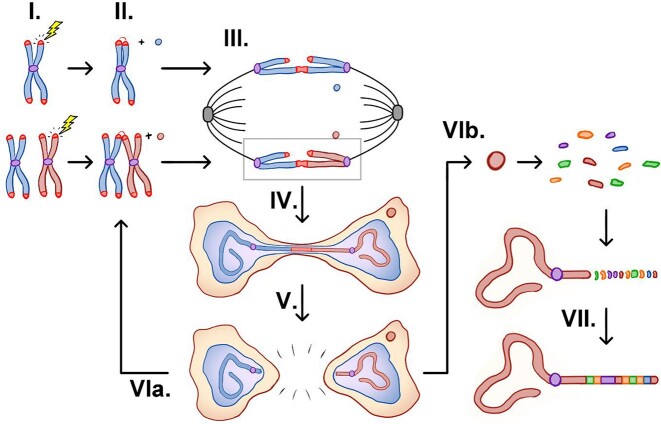
A diagram of the breakage-fusion-bridge cycle.

Genomic instability is one of the key hallmarks of cancer (1,2). Two main types of genomic instability are found in cancers: at the nucleotide level and at the chromosome level (3). The latter is referred to as chromosome instability (CIN), which has been observed in a wide variety of tumors (4,5). Though the underlying mechanisms of CIN are complex, the breakage-fusion-bridge (BFB) cycle is considered one of the major drivers of CIN (4–6). Dr Barbara McClintock initially proposed the BFB cycle in the late 1930s by observing the cytogenetic changes in maize chromosomes after treatment with ionizing radiation (IR) (7,8). The current model for the BFB cycle proposes that the cycle starts with the formation of a dicentric chromosome, which is induced by one or more double-stranded DNA breaks (DSBs), frequently taking place at telomeres (Figure 1, steps I and II). The piece(s) of chromosome detached from the main one can be temporarily compartmentalized in a micronucleus. If the dicentric chromosome is left unresolved and remains intact when the cell progress to anaphase, the dicentric chromosome then becomes an anaphase bridge when the two sets of chromosomes are pulled towards the opposing centrosomes by the mitotic spindles (Figure 1, step III) (9–11). If the anaphase bridge fails to be resolved and persists during cytokinesis, it manifests as an intercellular chromosome bridge (Figure 1, step IV) (12,13). The intercellular chromosome bridge needs to be severed before the generation of two fully separated daughter cells (Figure 1, step V). If the broken chromosomes remain unrepaired in the daughter cells, they can potentially fuse with another broken chromosome thereby forming a new dicentric chromosome (Figure 1, step VIa), and thus trigger the next BFB cycle. Alternatively, the broken chromosome can be healed via chromothripsis or some other mechanism(s) by re-incorporating the chromosome fragment protected in the micronuclei into the main chromosome and terminating the BFB cycle (Figure 1, steps VIb and VII) (5). The BFB cycle plays a crucial role in promoting tumorigenesis and tumor evolution. For example, various solid tumors manifest many features of the BFB cycle occurrence (6). Most recently, using cultured cancer cell lines, Shoshani *et al.* showed that the BFB cycle and chromothripsis contribute to the rapid tumor evolution, leading to resistance to chemotherapy (14).

Though the concept of the BFB cycle was proposed >80 years ago (7,8), elucidation of the detailed mechanism of the BFB cycle has been challenging because naturally occurring dicentric chromosomes and resultant intercellular chromosome bridges in normal mammalian cells are rare events, which are too low for consistent detection, quantification, and characterization (15,16). In addition, the genetic loci where DSBs can occur and subsequently lead to the formation of a dicentric chromosome are difficult to identify. Intriguingly, in cells with dysfunctional telomeres, telomere fusion-induced dicentric chromosomes are drastically increased (17–19). Therefore, these cells have been used to investigate the nature of dicentric chromosome formation and the related DNA repair processes. For example, studies using yeast and mammalian cells with dysfunctional telomeres have implicated both canonical non-homologous end joining (cNHEJ) as well as alternative non-homologous end joining (altNHEJ) in promoting the formation of telomere fusion-induced dicentric chromosomes (15,18,20,21). AltNHEJ has been renamed microhomology-mediated end joining (MMEJ) or Polθ-mediated end joining (TMEJ) (22).

In particular, inducing telomere dysfunction by perturbing the function of the Shelterin complex has been an important tool of obtaining information on chromosome fusion and breakage. TRF2 is a key component of Shelterin that binds and protects the integrity of telomeres (19). Using an inducible dominant-negative TRF2 (TRF2-DN) model (17), two groups demonstrated recently that cytoplasmic nucleases such as TREX1 and the actomyosin-dependent physical stretching can facilitate the breakage of the intercellular chromosome bridges (12,13). Using the TRF2-DN cellular model, multiple groups have also implicated chromothripsis and kataegis as ways to heal the broken chromosome(s) in the daughter cells (12,13,23). Additionally, Umbreit *et al.* have shown that depletion of a component of condesin using siRNA, treatment of cells with low-dose topoisomerase II inhibitor ICF-193, and CRISPR/Cas9-mediated cleavage of telomeres of chromosome 4 also recapitulate certain features of the BFB cycle (13).

Even though tremendous progress has been made in recent years to elucidate the detailed mechanism of the BFB cycle, many critical questions still need to be answered. For example, what type of genomic stress is capable of inducing the DSBs in Step I of the BFB cycle? To what extent are additional DSB repair pathways involved in the formation of dicentric chromosomes and intercellular chromosome bridges? Additionally, what is the molecular and structural nature of the intercellular chromosome bridge? Here, we first showed that targeting the nuclease-dead *Streptococcus pyogenes* Cas9 (dCas9) to telomeres with a telomeric single-guide (sg) RNA, or sgTelo, induces robust replication stress and DNA damage, specifically at telomeres. Remarkably, in the dCas9/sgTelo cells, we observed a drastic increase in the formation of chromosome end fusions, dicentric chromosomes, anaphase bridges, and intercellular telomeric chromosome bridges (ITCBs). Through this, we firmly established that the dCas9/sgTelo system can be used as a novel BFB cycle cellular model. Utilizing this model, we then investigated the mechanism of multiple steps of the BFB cycle. We not only confirmed the previous findings that cNHEJ promotes the formation of dicentric chromosomes, but further demonstrated that homology-dependent repair (HDR), including the break-induce replication (BIR), can also promote the formation of ITCBs. Most intriguingly, we found that TMEJ suppresses the formation of ITCB. Furthermore, we showed that when TMEJ is inhibited, cells upregulate HDR and downregulate cNHEJ to promote the formation of ITCB. Finally, we uncovered several interesting molecular and structural features of the ITCB through imaging. Collectively, our data shed new light on the detailed mechanisms of the BFB cycle, which will have an important impact on our understanding of tumorigenesis, tumor evolution, and drug resistance.

## Materials and methods

### Cell lines and tissue culture

U2OS-derived dCas9/sgTelo and dCas9/sgNS cell lines were generously provided by Dr Erik Sontheimer (University of Massachusetts Chan Medical School) (24). The sequences for sgNS and sgTelo are: gAATCTCGCTTATATAACGAG and gTTAGGGTTAGGGTTAGGGTT respectively. All cells are grown in DMEM (Corning) supplemented with 10% fetal bovine serum (Bio-techne), penicillin (Corning), and streptomycin (Corning), at 37 °C in a humidified incubator with 5% CO_2._ To induce the expression of dCas9-mCherry-APEX2, cells are grown in media containing 2 μg/ml doxycycline (Thermo Fisher) and 250 nM Shield1 (Takara Bio) for 21 h.

### Immunofluorescence

dCas9/sgTelo and dCas9/sgNS cells were seeded onto coverslips and induced with doxycycline and shield1 for 21 h before fixation. Cells were either then fixed with 3% paraformaldehyde containing 2% sucrose for 10 min, followed by treatment with Triton X-100 solution on ice for 5 min, or treated with Triton X-100 solution on ice for 5 min then fixed with 3% paraformaldehyde containing 2% sucrose for 10 min (pre-extraction). Cells are stained with primary antibodies and the respective Alexa-488 (Invitrogen) and Alexa-546 (Invitrogen) conjugated secondary antibodies. All the antibodies used for IF are listed in Supplementary Table S4. SlowFade Gold DAPI (S36938) was used to stain the DNA and the DNA knots on ITCBs. Images were collected by using an Olympus upright Fluorescent Microscope or Axio Observer.Z1/7 (Zeiss Microimaging GmbH, Jena Germany). Images were processed using Adobe Photoshop or ZEN 2.5 pro.

### ImmunoFISH

Immunofluorescent and telomere fluorescent *in situ* hybridization was performed as previously described (Pan X., 2017). The telomere PNA probe (TelC) was purchased from PNA Bio (F1004). Images were collected by using an Axio Observer.Z1/7 (Zeiss Microimaging GmbH, Jena Germany).

### Single molecule analysis of replicated DNA (SMARD)

The SMARD assay was performed essentially as described previously (25). Briefly, dCas9/sgNS and dCas9/sgTelo cells were sequentially labeled with 30 μM IdU (4 h) and 30 μM CldU (4 h). DNA isolation and processing for SMARD were as described previously (25). Following Pme I digestion, telomeric DNA fragments ranging from 160 to 200 kb were resolved by PFGE, identified by Southern blot, and then isolated. The Pme I-digested DNA was stretched on microscope slides coated with 3-aminopropyltriethoxysilane (Sigma). After stretching, the DNA was denatured in alkali-denaturing buffer (0.1 N NaOH in 70% ethanol and 0.1% β-mercaptoethanol) for 12 min and fixed by addition of 0.5% glutaraldehyde for 5 min. Telomeric DNA was identified by hybridization with a Biotin-OO-(CCCTAA)4 PNA probe (TelC, Biosynthesis) followed by incubation with Alexa Fluor 350-conjugated NeutrAvidin (Molecular Probes), followed by two rounds of incubation first with a biotinylated anti-avidin antibody (Vector) and then with the Alexa Fluor 350-conjugated NeutrAvidin. Incorporated halogenated nucleotides were detected with a mouse anti-IdU monoclonal antibody (BD) and a rat anti-CldU monoclonal antibody (Accurate) followed by Alexa Fluor 568-conjugated goat anti-mouse (Invitrogen) and Alexa Fluor 488-conjugated goat anti-rat secondary antibodies (Invitrogen). An antibody recognizing the single-stranded DNA was purchased from Millipore (anti-DNA antibody, clone 16–19, MAB3034).

### EdU labeling for DNA synthesis detection

To visualize DNA synthesis, induced dCas9/sgTelo, and dCas9/sgNS cells on coverslips were incubated with 10mM EdU for 15min or 30 min prior to fixation. Cells on coverslip were cooled on ice rinsed once with 1x PBS, treated with pre-extraction buffer (0.1% Triton X-100, 20 mM HEPES–KOH pH 7.9, 50 mM NaCl, 3 mM MgCl_2_, 300 mM sucrose) for 5 min on ice, rinsed once with 1 × PBS, fixed with 3% PFA and 2% sucrose for 15 min at room temperature and cold methanol for 10 min in −20°C. Cells were then permeabilized in PBS with 0.5%Triton X-100 for 3 min, and blocked with block solution (1 × PBS containing 0.05% Tween-20 and 3% BSA) for 1h at RT. The Click-iT® EdU Imaging Kit (Cat. Nos C10337) was used to detect the EdU. Images were collected by using an Axio Observer.Z1/7 (Zeiss Microimaging GmbH, Jena Germany).

### Metaphase spread assay

For metaphase spread preparations, cells were plated at 1 M cells per 10 cm plate on Day 1. On Day 2, cells were induced with doxycycline and shield1 as previously described. For inhibitor studies, various DNA repair enzyme inhibitors were added at specified concentrations overnight (approximately 6 hours after induction). On Day 3, KaryoMAX Colcemid solution (Gibco, Catalogue No. 15212012) was added to the media to achieve a final concentration of 0.1 μg/ml for 2.5 h. Cells were subsequently gently washed with PBS once, detached with 1mL of 0.25% trypsin and collected in media. Cells were centrifuged and pellet was washed with PBS and resuspended in 0.5 ml PBS. PBS was removed and cells were resuspended in 5 ml pre-warmed 37°C 0.075 M KCl solution. Cells were incubated for 15 min at room temperature. 1 ml of ice-cold fixative (3:1 volume of methanol:glacial acetic acid) was added dropwise and cells were incubated for an additional 10 min at room temperature. Cells were centrifuged for 5 min at 1000 rpm and KCl solution was removed except for 0.5 ml. Cells were resuspended in remaining KCl and additional 1mL of fixative was added while cells were vortexed at medium speed. An additional 8mL of fixative was added at high speed to ensure fixation. Cells were centrifuged again and resuspended in 200–300 μl of fresh fixative. Resuspended cells were dropped from several inches above the end of a dried tilted slide. Slides were airdried and mounted with DAPI. Metaphases were identified and imaged using a Axio Observer.Z1/7 (Zeiss Microimaging GmbH, Jena Germany) under 63× with oil immersion.

### Anaphase bridge assay

For analysis of anaphase bridges, cells were plated on coverslips one Day 1. On Day 2, cells were induced with doxycycline and shield1. On Day 3, Ro-3306 (Selleck, Catalog No. S7747) was added to the media for a final concentration of 7 μM and incubated at 37°C for 6 h. After incubation, cells were washed three times with pre-warmed media and the specified DNA repair enzyme inhibitors were added for 1 h and 15 min. Coverslips were then washed with PBS once and fixed at room temperature in 3% paraformaldehyde and 2% sucrose solution. Cells were washed again and mounted onto slides using DAPI solution. Anaphases were identified and imaged using a Axio Observer.Z1/7 (Zeiss Microimaging GmbH, Jena Germany) under 63× with oil immersion.

### Time-lapse imaging of live cells

Time-lapse imaging of live cells was done for 25 h with a time interval of 30 min between time points on a fully automated DVCore microscope (Leica-microsystems), using a 60× oil objective (N.A. 1.42) a chamber heated to 37°C and a Cascade2 EMCCD camera.

### siRNA transfection

Cells are transfected twice on consecutive days using 50 nM siRNA with RNAiMAX (Invitrogen) following the protocol recommended by the manufacturer. The sequence and order information for all the siRNA are listed in Supplementary Table S5.

### Long-term viability assay

4000 cells/per well are seeded in 12 well plates. Cells are induced the following day with media containing doxycycline and shield1. After 7–10 days of growth, cells are fixed using a methanol/acetic acid solution and stained with 1% crystal violet solution.

### Single-molecule telomere assay via optical mapping (SMTA-OM)

#### DNA extraction and purification

Cells were first embedded in gel plugs with approximately 1 million cells per gel plug (BioRad no. 170-3592). The high molecular weight genomic DNA was extracted and purified using the Bionano Genomics SP kit.

#### DNA labeling

Following the manufacturer's instructions, a DLS labeling kit (Bionano Genomics) labeled 750 ng of genomic DNA. A labeling mix comprised Direct Labeling Enzyme 1 (DLE-1), 1× DLS reaction buffer, and DL green fluorophore-labeled nucleotide mix. The labeling mix was added to the genomic DNA, gently mixed via a wide bore pipette, and then incubated at 37°C for 2 h. Following the incubation, membrane dialysis was used to remove the unwanted fluorescent dyes, proteins, and salts. Membrane dialysis was done at room temperature for approximately 2 h in the dark to protect it from the light. Dialyzed DNA was then recovered using a 100 nm hydrophilic membrane (EMD Millipore, VCWP04700) and quantified with a Qubit device.

The second part of the labeling procedure focuses on labeling the telomeres. Guide RNA (gRNA) consisting of 0.5 μM tracrRNA (IDT) and 50 μM crRNA was gently mixed via pipetting up and down and annealed on ice for 30 min. 25 pmol gRNA was incubated with 200 ng Cas9D10A nicking enzyme and 1× NE Buffer 3.1 at 37°C for 15 min. Next, 300 ng of the previously DLE-1 labeled genomic DNA was added to the Cas9D10A mixture for the one-hour nicking reaction at 37°C. After nicking, 200 nM red fluorophore-labeled nucleotides (ATTO647-dUTP, dATP, dGTP, dCTP) were incorporated into telomeres by 5 U Taq DNA polymerase at 72°C for 1 h in 1× Thermopol buffer (New England Biolabs). The nick-labeled sample was treated with proteinase K (QIAGEN) at 50°C for 30 min. Finally, to prepare the DNA for nanochannels, a staining mix of flow buffer, DTT and YOYO-1 (a blue-colored dye that labels the backbone of DNA, Bionano Genomics DLS kit) was prepared according to the manufacturer's instruction before adding it to the sample. The sample combined with the staining mix was incubated at room temperature overnight.

#### Imaging with Saphyr

A Bionano Saphyr G1.2 chip was loaded with the labeled DNA sample and imaged using a ‘dual-labeled sample’ scheme incorporated in the Saphyr software. Images were taken in the following order: first, the red fluorescent labeling with the 637 nm laser, then the green fluorescent labeling with the 532 nm laser, and finally, the blue fluorescent DNA backbone YOYO-1 staining with the 473 nm laser.

#### Genome assembly

*De novo* genome assembly was done using the software developed by Bionano Genomics. Consensus maps were generated by de novo assembling individual DNA molecules followed by alignment to the hg38 human reference genome.

#### Telomere analysis

CMAP, XMAP and BNX files were generated after *de novo* genome assembly. The CMAP files contain both the red and the green label information. The XMAP files contain only the information on the green labels. Molecules matching the expected green labeling patterns were extracted from the BNX and CMAP files. Raw molecule images were extracted using in-house software based on their locations in the nanochannels. The red telomere labels were easily distinguishable from the green labels in the subtelomeric region. Telomeres were then analyzed and measured using the ImageJ software. The ferret diameter tool was used to capture the length of telomeres in pixels and intensities before converting all the measurements to kilobase (kb) following the previously established procedure (26,27).

#### Inhibitor treatment assay

All small molecule inhibitors used in this study are listed in the Supplementary Table S6. For all small molecule inhibitor treatments, cells are plated on day 1. On day 2, the dcas9/sgTelo expression is first induced with doxycycline/shield1 for 6 hours. These cells are then treated with the chosen concentrations of each inhibitor for 15 h. After 21 h from the initial induction, cells were fixed, stained and imaged.

#### Immunoblotting

Cells are collected and lysed directly in a sample buffer, followed by sonication for 30 s. Equal volumes of lysate were loaded for immunoblotting. Antibodies used for immunoblotting: Actin (Santa Cruz, sc-1616); BLM (Bethyl, A300-110A and A300-120A), BRCA1 (EMD/Calbiochem, OP92); BRCA2 (EMD/Calbiochem, OP95), Rad51 (Santa Cruz, sc-8349); Rad52 (Santa Cruz, sc-365341); POLD3 (Abcam, 182564).

### Immunostaining and super-resolution imaging (STORM)

dCas9/sgTelo cells were seeded at a density of 3.5 × 10^5^ on coverslips in 6-well plates. The next day, cells were induced for 21 h with shield1 and doxycycline. Immediately after, cells were fixed with 3% PFA and 2% sucrose solution, washed with PBS, and permeabilized in a Triton X-100 solution. Primary antibody staining was done with RPA32-pS4pS8 (Bethyl, A300-245A), TRF1 (abcam, ab10579) and BLM (Bethyl, A300-110A). Secondary staining was done with anti-rabbit Alex Fluor 647 (Thermo Fisher, A32733), and the endogenous mCherry signal was boosted with anti-alpaca Alexa Fluor 568 RFP-booster (Chromotrek, rb2AF586). Unbound antibodies were washed off, cells were treated with 2% PFA and 3% sucrose solution for 10 minutes and again washed with PBS.

Super-resolution image acquisition was done with a Nanoimager super-resolution microscope (Oxford Nanoimaging Ltd, Oxford, UK), which was used with an Olympus 1.4NA 100 × oil immersion super apochromatic objective. Initial calibration was done using 0.1 μM Tetraspeck beads (Thermo Fisher). Cells were flushed with blinking induction buffer containing glucose, glucose oxidase, catalase, and β-mercaptoethanol prior to every imaging session. Excitation of Alexa Fluor 647 and RFP-boosted mCherry fluorophores was done using lasers set at 640 and 568 nm, respectively. Initially, the 568 nm laser was used at 3–4% power for an overview scan of a partial region of the coverslip to identify bridges. Following identifying a bridge of interest, multi-image acquisition was set up to take images at 30 ms/frame for each wavelength as a series of 5000 or 10 000 frames. Excitation at 640 nm was done first, followed by at 568 nm to limit potential photobleaching. Following image acquisition, initial 50–100 frames were filtered out, and the remaining were used for analysis.

### Detection and quantification of mitotic cells

To assess the population of mitotic cells, cells were seeded and treated using either small-molecule inhibitors or siRNA treatment as detailed above. After 21 h of induction, cells were trypsinized and collected. Media and PBS washes were collected as well. Cells were prepped using reagents from Intracellular Flow Cytometry Kit (Cell Signaling, Catalog No. 13593). Fixation was performed using 4% formaldehyde for 15 min at room temperature, followed by centrifugal washing with 1× PBS to remove excess fixative. Permeabilization involved slowly adding ice-cold 100% methanol to cells, gently vortexing to achieve a final concentration of 90% methanol and incubating on ice for at least 10 minutes. For immunostaining, cells were washed in excess 1X PBS to remove methanol and then resuspended in antibody dilution buffer with phospho-Histone H3 Alexa Fluor 488 (Ser-10) (1:50; Cell Signaling, Catalog No. 3465,) and incubated for 1 hour at room temperature. Cells were then stained with propidium iodide (25 μg/ml) and RNase A (200 μg/ml) solution for 30 min, resuspended in PBS and analyzed using a C6 flow cytometer under FL1 and FL2 lasers.

## Results

### Targeting dCas9 to telomeres induces replication stress, DNA damage and micronuclei formation

**Figure 2.**
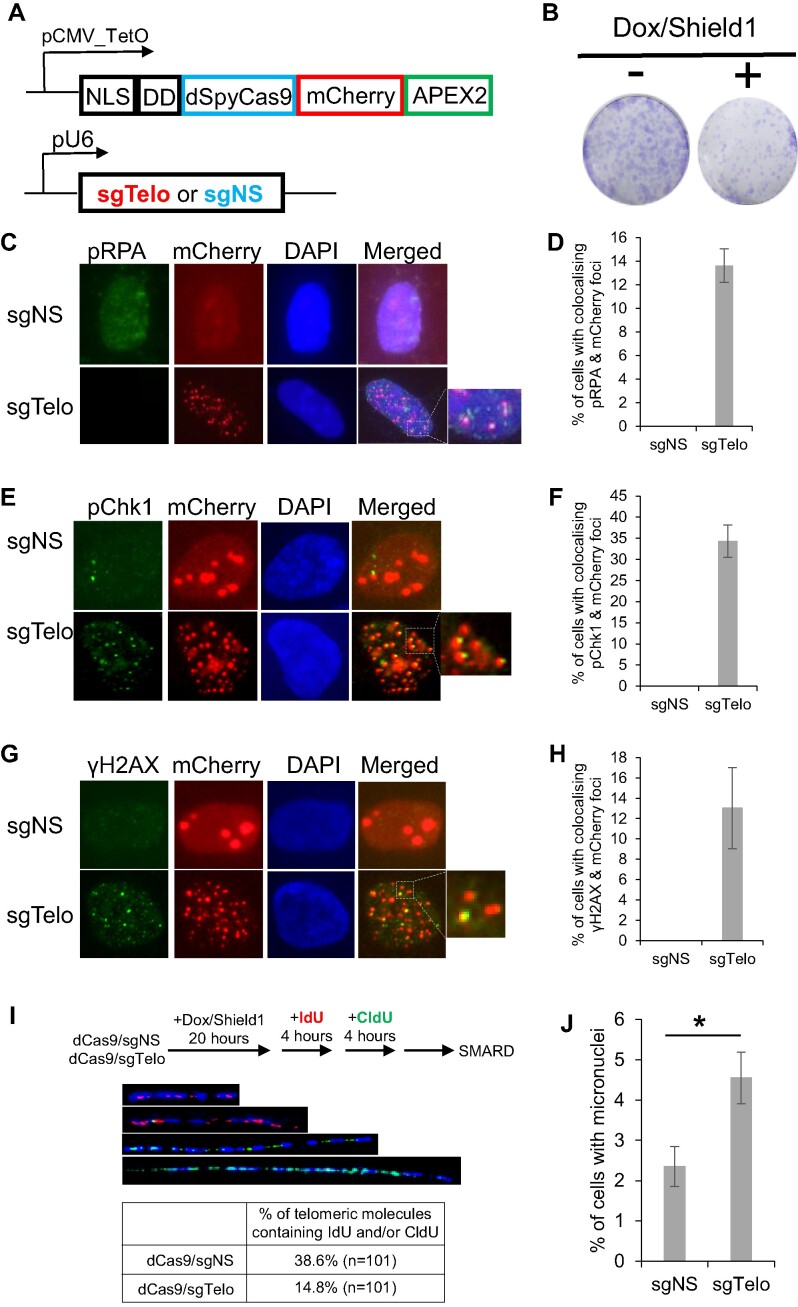
Targeting dCas9 to telomeres blocks DNA replication and induces pronounced replication stress and DNA damage. (**A**) A diagram of the inducible targeting of dCas9-mCherry-APEX2 specifically to telomeres. NLS: nuclear localization signal; DD: Shield1-repressible degradation domain. The expression of dCas9-mCherry-APEX2 is controlled by a doxycycline (Dox) inducible promoter (pCMV-TetO). dCas9-mCherry-APEX2 is further stabilized by adding Shield1, which inactivates the DD domain. sgNS: a non-specific single-guide RNA; sgTelo: a telomeric single-guide RNA. (**B**) Long-term viability assay comparing non-induced (-) and induced (+, with the addition of both Dox and Shield1) dCas9/sgTelo cells. (C–H) 21 h after the addition of Dox and Shield1, dCas9/sgNS and dCas9/sgTelo cells were fixed and stained with antibodies recognizing either phosphorylated RPA (labeled as pRPA in **C** and **D**), or phosphorylated Chk1 (labeled as pChk1 in **E** and **F**) or γH2AX (**G** and **H**). All nuclei were also stained with DAPI. More than 200 cells were counted. Error bars are standard deviations from three independent experiments. (**I**) dCas9/sgTelo reduces the replication efficiency of telomeric DNA. dCas9/sgNS and dCas9/sgTelo cells were first treated with Dox and Shield1 for 20 hours and then sequentially pulse-labeled with IdU and CldU for 4 h each. SMARD was performed on telomeric DNA PmeI fragments isolated from these cells as detailed in Materials and Methods, with fragments ranging from 160 to 200 kb. The telomeric DNA molecules of variable lengths were identified by FISH using telomeric PNA probes (TelC, blue). The incorporated IdU and CldU were detected by indirect immunofluorescence with antibodies recognizing IdU (red) and CldU (green). Four representative telomeric molecules are shown. (**J**) Increased micronuclei formation in dCas9/sgTelo cells. More than 1000 cells were counted. Error bars are standard deviations from two independent experiments. Standard two-tailed Student's *t*-test: *P < 0.05.

Previously, dCas9 was shown to be a targetable roadblock for DNA replication *in vitro* (28). In a separate study, Sontheimer and his colleagues developed a doxycycline (Dox)-inducible system in U2OS cells to identify proteins near a well-defined genomic locus, called C-BERST (dCas9-APEX2 biotinylation at genomic elements by restricted spatial tagging, Figure 2A) (24). They designed two sgRNAs, sgAlpha, which targets the dCas9-APEX2 to centromeres, and sgTelo, which targets the dCas9-APEX2 to telomeres. An sgRNA targeting non-specific DNA sequences, or sgNS, was used as the negative control. They identified many well-known centromere and telomere-associated proteins through mass spectrometry. Intriguingly, they also identified many DNA damage response (DDR) proteins at both centromeres and telomeres. Among them are BLM, BRCA1 and FANCM, which we have shown previously to suppress the replication stress at Alternative Lengthening of Telomere (ALT) telomeres (29). Intrigued by the results of this study, we hypothesized that targeting the dCas9-APEX2 protein to telomeres impedes the progression of the replisome, leading to stalling of the replication forks at telomeres and activation of the replication stress response and DDR pathways. An additional feature of the C-BERST system is the inclusion of a mCherry marker within the dCas9-APEX2 fusion (Figure 2A), which can be used to visualize the locations of dCas9/sgRNA in both fixed and live cells.

To test our hypothesis, we first confirmed that a 21-h treatment of dCas9/sgTelo cells with Dox and Shield1 led to an increase of mCherry foci that colocalize with both TelC foci and TRF1 foci (Supplementary Figure S1A). TelC is a telomere-specific PNA probe that detects telomeric DNA through *in situ* hybridization (29). TRF1 is part of the Shelterin complex and is also widely used as a marker for telomeres (19). Therefore, mCherry can also be used as a reliable telomere marker in the dCas9/sgTelo cells. Targeting dCas9-mCherry-APEX2, abbreviated as dCas9, to telomeres compromised the long-term viability of the dCas9/sgTelo cells (Figure 2B) and induced robust replication stress and DNA damage at telomeres as indicated by the increased colocalization of mCherry foci with phosphorylated Serine-4/Serine-8 RPA (pRPA) foci, phosphorylated Serine-345 of Chk1 (pChk1) foci, and with γH2AX foci, respectively (Figure 2C–H). The patchy red signals seen in the dCas9/sgNS cells are likely due to the aggregation of mCherry in the nuclei (Figure 2C and E). In addition to pRPA, pChk1 and γH2AX, many other well-known DDR proteins, including BLM and RPA32, can also be detected in the mCherry foci (Supplementary Figure S1B–G). To examine whether dCas9/sgTelo directly affects DNA replication at telomeres, we performed the Single Molecule Analysis of Replication DNA (SMARD) assay (29). Indeed, we observed a 2.6-fold decrease in the replication efficiency of telomeric DNA in the dCas9/sgTelo cells compared to dCas9/sgNS cells (Figure 2I). Consistent with the heightened replication stress and increased DNA damage at the telomeres of the dCas9/sgTelo cells, we also observed a two-fold increase in micronuclei formation (Figure 2J).

Taken together, our data strongly indicate that targeting dCas9 to telomeres physically impedes the progression of DNA replication machinery at telomeres. This leads to stalled replication forks, the activation of a robust replication stress response, increased DNA damage, increased micronuclei formation and decreased cell viability.

### dCas9/sgTelo promotes the formation of chromosome end fusions

**Figure 3.**
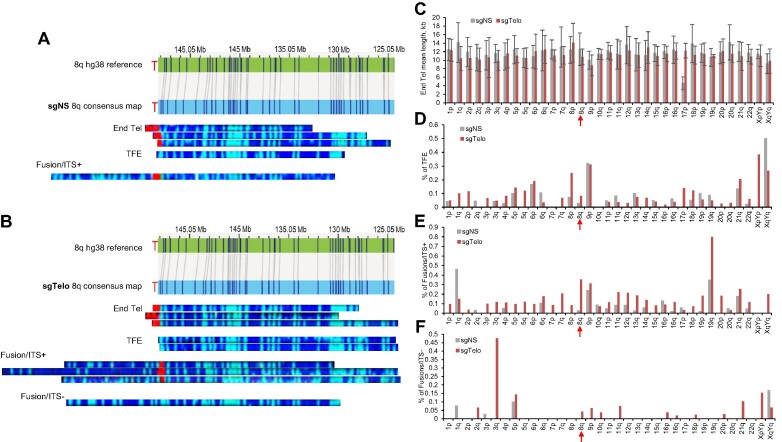
Genome-wide analysis of telomeres/chromosomes ends at the individual chromosome arm level. dCas9/sgNS and dCas9/sgTelo cells were treated with doxycycline and Shield1 for 21 hours and then were used for SMTA-OM analysis. (A and B) Representative optical mapping images of chromosome arm 8q from the dCas9/sgNS cells (**A**) and dCas9/sgTelo cells (**B**). (C–F) The SMTA-OM profiles of 35 chromosome arms, including End Tel mean length (**C**), percentage of TFE (**D**), percentage of fusion/ITS+ (**E**) and percentage of fusion/ITS– (**F**). Red arrows highlight chromosome arm 8q.

Having established that targeting dCas9 to telomeres induces a robust replication stress response and DNA damage, we sought to thoroughly characterize the genome-wide changes at telomeres and chromosome ends in the dCas9/sgTelo cells using the recently developed Single-Molecule Telomere Assay via Optical Mapping (SMTA-OM) (26,27,30,31). The principle of three-color SMTA-OM and some of its readouts are illustrated in Supplementary Figure S2. Briefly, long DNA fibers are carefully extracted and purified from cultured cells. The long DNA fibers (>150 kb) are first tagged with a green fluorescent label by the DLE-1 enzyme (Supplementary Figure S2A) (32). Telomeres are then specifically labeled with a red fluorescent label by CRISPR/Cas9 (Supplementary Figure S2A) (26). DNA backbones are stained with a blue DNA dye, YOYO-1. The DNA fibers are linearized through nanochannels and imaged. The following telomere/chromosome end features can be obtained from a single SMTA-OM assay (Figure 3A and B, Supplementary Figure S2B). (i) End telomere (End Tel) refers to a DNA molecule that is embedded in a blue labeled DNA molecule and has a red signal (telomere) at the end of a specific chromosome arm, which is identified by the pattern of green labels matched to the hg38 reference genome. The intensity of the red signal is used to measure the length of a telomere of a specific chromosome arm. (ii) Telomere-free end (TFE) refers to a specific chromosome arm without any detectable red signal at its end. (iii) Fusion/ITS+, or ITS+: one side of a red signal is attached to an identifiable chromosome arm, while the other side of the red signal is also attached to a green-labeled DNA fragment. However, because the latter DNA fragment is too short, it cannot be unambiguously assigned to any specific chromosome arm based on the pattern of the green labels. (iv) Fusion/ITS-, or ITS-: one portion of the green-labeled DNA fragment can be assigned to a specific chromosome arm based on the pattern of the green labels, while the other portion cannot be assigned to any chromosome arm again because the DNA fragment is too short. Meanwhile, there is no detectable telomeric signal (RED) in between the two DNA fragments. Compared to other widely used telomere assays (33), such as telomere restriction fragment assay (TRF), quantitative fluorescent *in situ* hybridization (Q-FISH), and STELA, the SMTA-OM offers several unique advantages. (i) SMTA-OM can measure a broad telomere length range, from 100 bp to more than 100 kb. (ii) SMTA-OM can unambiguously assign a telomere to a specific chromosome arm. Therefore, it can monitor genome-wide telomere changes at the specific chromosome arm level and the single-molecule level. (iii) SMTA-OM can also detect the TFE of a specific chromosome arm induced by DNA damage in the subtelomeres. (iv) SMTA-OM can unambiguously assign one portion of the fused chromosome in ITS+ or ITS– to a specific chromosome arm.

Using the SMTA-OM, we comprehensively characterized the aforementioned telomere/chromosome end features of 35 chromosome arms by imaging approximately 30 molecules per arm for both dCas9/sgTelo and dCas9/sgNS cells. The optical mapping images of two representative chromosome arms, 8q and 3q, are shown in Figure 3A and B, and Supplementary Figures S3 and S4. The raw measurements of the 35 chromosome arms are included in the Supplementary Table S1. The number of single molecules for each arm and calculated mean lengths, standard deviations, and frequencies of end telomere, TFE, ITS+ and ITS− events per arm from both samples are summarized in Supplementary Table S2.

Regarding the length of End Tel, we observed significant changes for particular chromosome arms (Figure 3C, Supplementary Figure S5, and Table 1). For example, the length of telomeres of 14q showed a 17% increase (13 kb for dCas9/sgTelo and 11.1 kb for dCas9/sgNS), while those of 20q showed an 19% decrease (11.5 kb for dCas9/sgTelo and 14.2 kb for dCas9/sgNS), suggesting there are chromosome arm-specific changes. However, when considering all the chromosome arms examined together, the overall telomere length change of End Tel is statically insignificant between dCas9/sgTelo and dCas9/sgNS (11.3 kb for dCas9/sgTelo and 11.7 kb for dCas9/sgNS, Supplementary Figure S5 and Table 1). Regarding the TFEs, we observed a 60% overall increase in frequency within dCas9/sgTelo cells compared to the control (Figure 3D and Supplementary Figure S6, Table 1), suggesting that many chromosome arms in dCas9/sgTelo cells have lost most of their telomeres.

**Table 1.** Summary of overall changes at telomeres/chromosome ends of different chromosome arms

	End Tel mean (kb)	Fusion/ITS + mean (kb)	Fusion/ITS+ (%)	Fusion/ITS− (%)	TFE (%)
sgTelo	11.3	11.8	15.2 (161/1061)	4.0 (42/1061)	8.0 (86/1061)
sgNS	11.7	11.2	5.4 (59/1100)	0.36 (4/1100)	5.0 (55/1100)
P value	0.2480	0.8690	0.0001	0.0790	0.04

Notes: (1) The numbers in the parentheses in rows 2 and 3 indicate the molecules imaged and analyzed. (2) When calculating the average telomere length of End Tel (End Tel mean), DNA molecules with telomere free ends (TFE) were excluded.

When examining telomere fusions, we observed a 2.8-fold and a 11.1-fold increase for the fusion/ITS+ (Figure 3E and Supplementary Figure S7, Table 1) and fusion/ITS− (Figure 3F and Supplementary Figure S8, Table 1) in the dCas9/sgTelo cells compared to the dCas9/sgNS cells, respectively. Since fusion/ITS− is potentially formed from two TFEs, the drastic increase of fusion/ITS- suggests that certain chromosome arms in the dCas9/sgTelo cells have lost most of their telomeres, which is consistent with the increase of TFEs. Similar to the End Tel findings, there are notable changes for different chromosome arms. For example, the fusion/ITS+ increased by 4.9-fold for 8q (Supplementary Figure S7). For 3q, the fusion/ITS- increased from 0% in the dCas9/sgNS cells to 47.6% in the dCas9/sgTelo cells (Supplementary Figure S8). There are no overall changes in the mean length of telomeres in fusion/ITS+ (Supplementary Figure S9 and Table 1).

In summary, the SMTA-OM analysis demonstrates that in the dCas9/sgTelo cells, the heightened replication stress at telomeres induces dramatic telomeric and subtelomeric DNA damage, leading to increased TFEs, fusion/ITS+ and fusion/ITS−. Most importantly, we also observed pronounced chromosome arm-specific changes for all the SMTA-OM readouts, indicating that telomeres of different chromosome arms respond to replication stress differently. These results underscore the importance of monitoring the telomere/chromosome end changes at the individual chromosome arm level.

### dCas9/sgTelo promotes the formation of dicentric chromosomes and anaphase bridges

**Figure 4.**
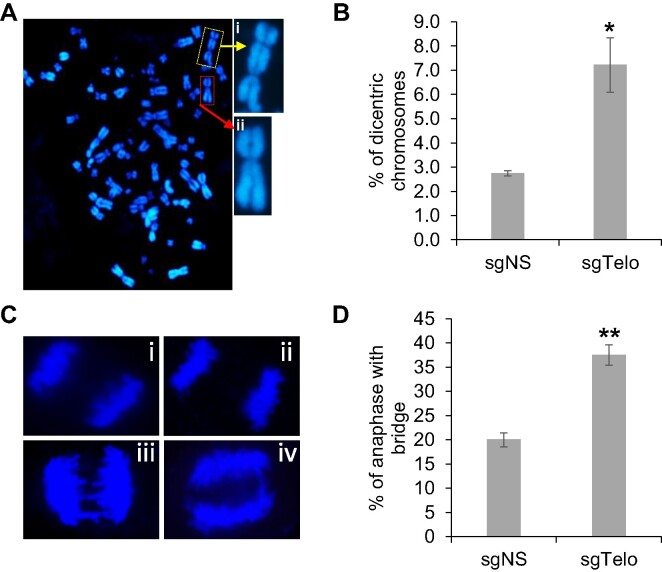
Targeting dCas9 to telomeres dramatically increases dicentric chromosomes and anaphase bridges. (**A** and **B**) dCas9/sgNS and dCas9/sgTelo cells were treated with doxycycline and Shield1 for 21 hours and then were used for metaphase spread analysis. (A-i) The representative image of a dicentric chromosome. (A-ii) The representative image of a normal monocentric chromosome. More than 2500 metaphase chromosomes were analyzed and qualified per condition. Error bars are standard deviations from three independent experiments. Standard two-tailed Student's *t*-test: *P < 0.05. (**C** and **D**) dCas9/sgNS and dCas9/sgTelo cells were treated with doxycycline and Shield1 for 21 h and then were used for anaphase bridge analysis. (C-i and C-ii) Two normal anaphase cells. (C-iii and C-iv) Two anaphase cells with bridges. Minimum 50 anaphase cells were analyzed and quantified. Error bars are standard deviations from three independent experiments. Standard two-tailed Student's *t*-test: *P < 0.05; **P < 0.01.

The drastic increase of fusion/ITS+ and fusion/ITS− observed from the SMTA-OM analysis suggests that they are likely derived from different types of dicentric chromosomes induced by DSBs generated within telomeres, or at the subtelomeres, or the combination of both. We performed metaphase spread assays to monitor the changes in dicentric chromosomes frequency directly. As shown in Figure 4A and B, we observed a 3-fold increase of dicentric chromosomes in the dCas9/sgTelo cells compared to the dCas9/sgNS cells. Consistent with the increase of dicentric chromosomes, we also observed a 2-fold increase of anaphase bridges in the dCas9/sgTelo cells compared to the dCas9/sgNS cells (Figure 4C and D), suggesting that the majority of the dicentric chromosomes remain unresolved and persist into the anaphase.

### dCas9/sgTelo promotes the formation of intercellular telomeric chromosome bridges

**Figure 5.**
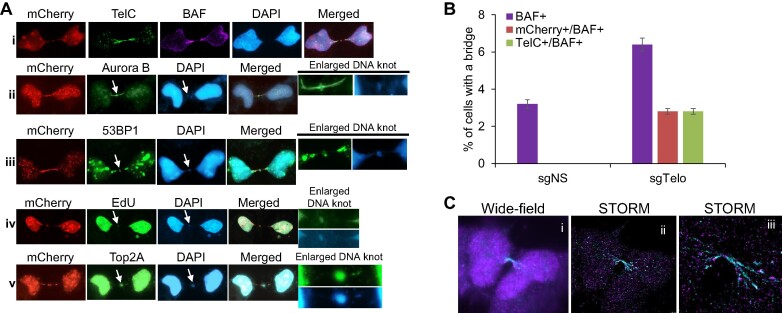
Targeting dCas9 to telomeres induces a drastic increase in intercellular telomeric chromosome bridges (ITCBs). (**A**) dCas9/sgTelo cells were treated with doxycycline and Shield1 for 21 h. Cells were then fixed and stained with either a PNA probe recognizing telomeres (TelC) or antibodies recognizing different proteins as indicated. For the EdU pulse labeling image (A-iv), EdU was added to the growth medium 30 min before fixation. (**B**) More than 3000 cells were analyzed and quantified. (**C**) dCas9/sgTelo cells were treated with doxycycline and Shield1 for 21 h. Cells were then fixed, stained with antibodies recognizing mCherry and were imaged with the STORM. (C-i) Wide-field imaging of an ITCB. (C-ii) STORM imaging of the same ITCB. (C-iii) Zooming image of the same ITCB.

Next, we investigated the cellular consequences of the drastically increased chromosome fusions, dicentric chromosomes and anaphase bridges in the dCas9/sgTelo cells. We reasoned that without successful resolution, the fused chromosomes could eventually lead to the formation of intercellular chromosome bridges during cytokinesis (step IV, Figure 1). Indeed, after imaging thousands of cells, we detected intercellular telomeric chromosome bridges (ITCBs), manifesting as two interphase cells with de-condensed chromosomes connected by a red/mCherry positive bridge, in ∼3% of the dCas9/sgTelo cells (Figure 5A and B). Previously, a non-specific double-stranded DNA binding protein, Barrier-to-Autointegration Factor (BAF), has been used as a marker for the intercellular chromosome bridges (12,13). Every ITCB we identified was also positive for BAF (Figure 5A-i). And when we stained the ITCBs with the TelC probe, all the mCherry bridges were also positive for TelC, confirming that the ITCBs indeed consisted of telomeric DNA. When the BAF marker and TelC probes were used to stain the control sgNS cells, we identified BAF + bridges at a frequency of 3%, but none of these bridges were TelC-positive or mCherry-positive (Figure 5B). Collectively, our data indicate that the formation of ITCB is a rare phenomenon in the non-stressed control cells. However, the induction of the dCas9/sgTelo triggers the formation of predominantly telomere-mediated fusions and subsequently ITCBs with telomeric DNA.

We then sought to carefully characterize the structure of the ITCBs and delineate staining patterns at the bridge body and the bridge bases. We observed that intriguingly, on approximately 86% of the ITCBs, we can detect a more intense DAPI-positive punctate structure in the middle of the ITCB, which we denote as a ‘DNA knot’ (Figure 5A-ii to A-v, Supplementary Figure S10). We also noted that approximately 83% of the BAF-positive intercellular chromosome bridges in the sgNS cells also contain a DNA knot, suggesting that the formation of the DNA knot is independent of whether the bridge is induced by telomeric fusion or non-telomeric fusion. Given the location of the DNA knot occurring between two daughter cells, we hypothesized this could be the location of the midbody. Indeed, 100% of the DNA knots stained positive for Aurora B (Figure 5A-ii), a commonly used marker for the midbody, indicating that the DNA knots are likely part of the midbodies. We also stained the ITCBs with an antibody recognizing 53BP1, a master regulator of DDR and DSB repair pathway choices (34,35). We not only detected 53BP1 at 70% of the DNA knots but also at 72% of the bridge bases and 30% of the bridge bodies (Figure 5A-iii and Supplementary Figure S10). To test whether there is active DNA synthesis at the DNA knots, we pulse-labeled dCas9/sgTelo cells with EdU for either 15 or 30 min prior to fixation (Figure 5-iv and Supplementary Figure S11). We observed positive EdU signals at 31% and 38% of the DNA knots, respectively, indicating active DNA synthesis in one third of the DNA knots. In addition, more than half of the DNA knots are also stained positive for Top2A, suggesting that there may be over-twisted or catenated DNA in the DNA knots (Figure 5A-v and Supplementary Figure S12).

In light of the discovery of DNA knots and the interesting staining patterns of 53BP1 and Top2A, we surveyed a panel of DDR proteins in the ITCB-forming cells. The DDR proteins surveyed so far manifest three distinct staining patterns: (i) DDR proteins, such as 53BP1, RPA32, cGAS, and FANCI, stain positive on the bridge body (Figure 5A-iii, Supplementary Figures S13A–C and N). Positive staining of RPA32 on the bodies of ITCBs suggests that there are single-stranded DNA (ssDNA) regions on the ITCBs. (ii) DDR proteins, such as 53BP1, γH2AX, and pChk1 stain positive on one or both bridge bases (Figure 5A-iii and Supplementary Figures S13D and E, N). (iii) DDR proteins, including 53BP1, FANCM, BLM and Rad51, localize in the middle of the bridges (Figure 5A-iii, Supplementary Figures S13F–M and N).

To gain further structural insights into the ITCBs, we also performed Stochastic Optical Reconstruction Microscopy (STORM). We observed that most ITCBs are branched and have a similar number of branches on both sides of the bridges (Figure 5C). We speculate that these branches reflect the number of dicentric chromosomes in the ITCBs. The average branches per ITCB are 2.846 (Supplementary Figure S14A and Supplementary Table S3), with an average length of 6.353 μm (Supplementary Figure S14B and Supplementary Table S3) and an average width of 0.285 μm (Supplementary Figure S14C and Supplementary Table S3). These data suggest that ITCBs may contain bundled dicentric chromosomes.

### Formation of an ITCB in the dCas9/sgTelo cells delays the completion of cytokinesis

**Figure 6.**
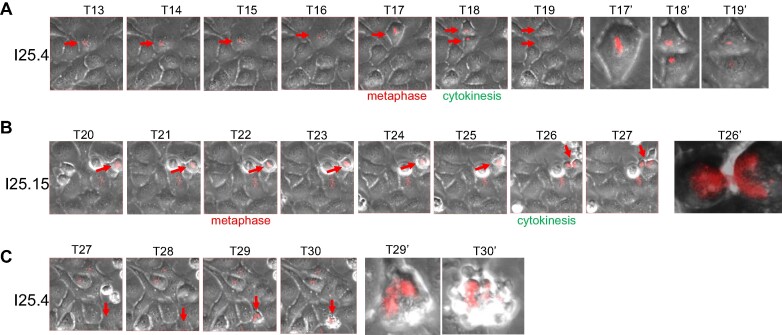
Time-lapse analysis of live dCas9/sgTelo cells. Asynchronous dCas9/sgTelo cells were pre-treated with doxycycline and Shield1 for 8 h. The cells were kept in doxycycline and Shield1 and monitored using a fully automated DVCore microscope (Leica-microsystems). Images were acquired every 30 minutes for 25 hours as indicated by the Tn. Tn’ is the enlarged view of the corresponding Tn. (**A**) The red arrows indicate a cell that expressed a low level of mCherry and manifested relatively normal cell cycle progression. (**B**) The red arrows indicate a cell that formed the intercellular telomeric chromosome bridge at T26. (**C**) The red arrows indicate a cell that underwent a mitotic catastrophe.

Next, we investigated the effects of the ITCBs on cell cycle progression in live cells by performing a time-lapse experiment. When the expression of mCherry is relatively low, the cell progresses through mitosis and cytokinesis without delay. It usually takes <30 min for all the chromosomes to align at the metaphase plate and complete cytokinesis and produce two daughter cells (Figure 6A, Supplementary Figures S15A and B, and Supplementary Movie S1). In stark contrast, in the cell that eventually forms an ITCB, the time between metaphase and completion of cytokinesis increases to between 90 and 120 min (Figure 6B, Supplementary Figure S15C, and Supplementary Movie S2). This suggests that the presence of an ITCB triggers the activation of the abscission checkpoint, thereby delaying the completion of cytokinesis to give the cell more time to resolve the ITCB. We also noted that many mCherry-positive cells failed to progress through the different stages of mitosis and eventually committed to cell death, vis-à-vis mitotic catastrophe (Figure 6C, Supplementary Figure S15D and Supplementary Movie S1).

In summary, we have shown that: (i) targeting dCas9/sgTelo to telomeres induces robust replication stress, specifically at telomeres (Figures 2C–F), which can lead to increased DNA damage (Figures 2G and H; step I, Figure 1); (ii) the induced telomeric DNA damage then serves as substrates for the formation of dicentric chromosomes (Figures 4A and B; step II, Figure 1); (iii) when the dicentric chromosomes remain unresolved and persist into anaphase, they manifest as anaphase bridges (Figures 4C and D; Step III, Figure 1); (iv) the unresolved anaphase bridges subsequently lead to prolonged mitosis, cytokinesis, and the formation of ITCBs (Figure 5; step IV, Figure 1). Taken together, our data firmly establish that the dCas9/sgTelo system can be used as a novel cellular model for the BFB cycle.

### cNHEJ, HDR and BIR promote the formation of ITCBs in the dCas9/sgTelo cells, while TMEJ inhibits them

**Figure 7.**
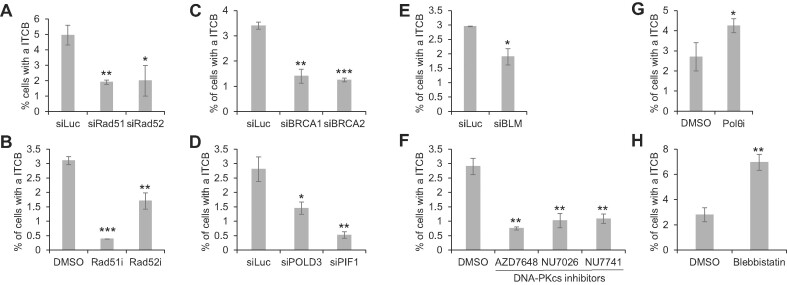
Multiple DNA double-stranded break (DSB) repair pathways regulate the formation of intercellular telomeric chromosome bridges. (**A, C, D, E**) dCas9/sgTelo cells were transfected with different siRNA twice. 24 h after the second transfection, cells were treated with doxycycline and Shield1 for 21 h before they were fixed and imaged. (**B**, **F**, **G**, **H**) dCas9/sgTelo cells were pre-treated with doxycycline and Shield1 for 6 h. Small molecule inhibitors targeting different proteins were then added to the growth medium. Cells were grown for another 15 h before they were fixed and imaged. More than 3000 cells were counted. Error bars are standard deviations from three independent experiments. Standard two-tailed Student's *t*-test: *P < 0.05, **P < 0.01, ***P < 0.005.

**Figure 8.**
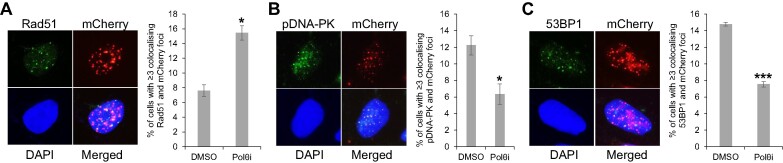
Inhibition of Polθ leads to increased HDR activity and decreased NHEJ activity. dCas9/sgTelo cells were pre-treated with doxycycline and Shield1 for 6 h. Polθ inhibitor was then added to the growth medium. Cells were grown for another 15 hours before they were fixed and stained with antibodies recognizing either Rad51 (**A**), phosphor-DNA-PKcs (**B**, pDNA-PK) or 53BP1 (**C**). More than 300 cells were analyzed. Error bars are standard deviations from two independent experiments. Standard two-tailed Student's *t*-test: *P < 0.05. **P < 0.01. ***P < 0.005.

Given the consistent detection of approximately 3% of the dCas9/sgTelo cells forming ITCBs (Figures 5A and B), we then used the ITCB formation as a functional readout of the BFB cycle. We investigated the role of different DSB repair pathways in their formation. Depending on the cell cycle stage and the availability of a source of homology, DSBs in eukaryotes can be repaired by either NHEJ or homology-dependent repair (HDR) (36). NHEJ consists of at least two subtypes, cNHEJ, which is dependent on Ku70/80, DNA-PKcs, and Ligase IV and TMEJ, which is dependent on Pol θ, XRCC1, and Ligase III (22). HDR also consists of a few sub-types, including double Holiday Junction (dHJ), synthesis-dependent strand-annealing (SDSA), and break-induced replication (BIR). Using either siRNA (Supplementary Figure S16) or small molecule inhibitors, none of which lead to pronounced impacts on the cell cycle profiles and mitotic population (Supplementary Figures S17 and S18), we demonstrated that inactivation of proteins involved in HDR (Rad51, Rad52, BRCA1 and BRCA2; Figures 7A–C), BIR (POLD3, PIF1 and BLM; Figures 7D and E), or cNHEJ (DNA-PKcs; Figure 7F) attenuated the formation of ITCBs. Meanwhile, the inactivation of Polθ (Figure 7G) promoted their formation. The corollary to this finding is that the normal presence of Polθ suppresses the formation of ITCBs, which is somewhat surprising. We wondered if inhibition of Polθ could have shifted the onus of DNA repair to the other available DSB repair pathways. Therefore, we further examined the activity of HDR and cNHEJ in the Polθi treated dCas9/sgTelo cells. Intriguingly, we found that inhibition of Polθ led to increased Rad51 foci and decreased phospho-DNA-PKcs (pDNA-PK) foci and 53BP1 foci (Figure 8). These results suggest that when TMEJ is blocked, telomeric breaks are preferentially repaired through HDR rather than cNHEJ to promote the formation of dicentric chromosomes, anaphase bridges, and ITCBs.

Finally, we investigated the mechanism of the breakage of ITCBs. A recent study by Pellman *et al.* suggests that the intercellular chromosome bridge is primarily broken via forces utilizing the actomyosin-dependent mechanical stretching (13). Consistent with their findings, inhibition of myosin II led to a 3-fold increase of ITCBs (Figure 7H), indicating that many of the ITCBs in the dCas9/sgTelo system are also broken off by the actomyosin-dependent mechanical stretching.

Collectively, our data demonstrate that multiple DSB repair pathways are implicated in forming dicentric chromosomes, anaphase bridges, and ITCBs. cNHEJ and HDR/BIR promote the formation of ITCBs, while the TMEJ suppresses their formation.

## Discussion

As predicted by McClintock’s original studies, chromosomes experiencing telomeric loss can undergo BFB cycle initiation. However, the precise conditions that create a cellular environment which enables telomere fusion are still unknown. Given that the BFB cycle has such significant implications for tumorigenesis and tumor evolution, understanding it in greater detail is of the utmost importance and this requires additional cellular models (4,37). In this study, we established the dCas9-mCherry-APEX2/sgTelo system as an additional cellular model for the BFB cycle, which will complement nicely the current BFB cycle models. First, we demonstrated that targeting dCas9 to telomeres impedes DNA replication and induces pronounced and localized replication stress. This replication stress then induces DNA damage and DSBs at telomeres (step I of the BFB cycle, Figure 1), which subsequently lead to the formation of dicentric chromosomes (step II of the BFB cycle, Figure 1), anaphase bridges (step III of the BFB cycle, Figure 1), and intercellular chromosome bridges (step IV of the BFB cycle, Figure 1). In addition, we also demonstrated that breakage of the ITCBs in the dCas9/sgTelo system is partially due to the actomyosin-dependent mechanical stretching, consistent with the previous finding from Pellman's group (13). Using this newly established BFB cycle model, we showed that multiple DSB repair pathways are implicated in the formation or suppression of dicentric chromosomes, anaphase bridges, and the ITCBs. Specifically, we found that cNHEJ, HDR and BIR promote the formation of ITCBs while TMEJ suppresses them. Finally, utilizing various molecular and cellular assays, we uncovered many novel features of the intercellular chromosome bridges. These include discovering the DNA knots in the middle of many ITCBs and the active DNA synthesis/repair at many DNA knots. The findings presented here will profoundly impact the understanding of how the BFB cycle affects tumorigenesis, tumor evolution, and drug resistance. Our dCas9/sgTelo model is established in U2OS, a well-known ALT-positive cancer cell line. Whether the dCas9/sgTelo technology can induce a robust BFB cycle in the telomerase-positive cell line or the non-transformed cell line warrants further investigation. In addition, since APEX2 is also part of the dCas9 fusion protein, the reactive oxygen species produced by the APEX2 *in vivo* may potentially also contribute to generating telomeric DNA breaks and other observed phenotypes in our system.

### dCas9/sgRNA as a versatile and locus-specific tool for investigating replication stress in vivo

DNA damage can be induced by various endogenous or exogenous sources (36,38). Replication stress is one of the most common endogenous sources of DNA damage, especially at those difficult-to-replicate (DTR) genomic loci, including centromeres, common fragile sites, and telomeres, which contribute to the development of many human diseases including cancer (39–41). The source of the replication stress at these sites can include factors such as dysregulated DNA repair, nucleotide imbalances, fork barriers etc. Replication fork barriers can be induced by both exogenous and endogenous sources and are of particular interest in the context of this study. Exogenous sources include exposure to UV radiation, or compounds such as cross-linking agents (e.g. mitomycin C and cisplatin), or topoisomerase inhibitors (i.e. camptothecin) and others. Endogenous fork barriers can include secondary DNA:RNA hybrid structures or DNA structures like G-4 quadruplexes.

Much of the research done on sources of replication stress and cellular response to replication stress relies on the exogenous replication stress inducers (RSI). The disadvantages of using these exogenous RSIs include (i) inducing DNA damage via non-DNA replication-related mechanisms and (ii) indiscriminately blocking DNA replication at many genomic regions/loci. This in turn has limited the understanding of effects of specific localized replication stress responses, making new replication stress models of utmost importance. To address these limitations, a few novel endogenous and locus-specific replication fork blocks (RFBs) via strong protein-DNA interactions, including LacO-LacI, TetO-TetR and Tus-Ter, have been developed in mammalian cells (42–44). In addition, we recently demonstrated that depletion of FANCM in cells that adopted the ALT pathway induces a pronounced replication stress response at telomeres, and we thus proposed that the FANCM deficiency-induced replication stress at ALT telomeres (MR-SAT) can be used as a telomere-specific DNA replication perturbation model (29,45). Using the MR-SAT system, we uncovered a novel DNA damage checkpoint function of POLDIP3 (46). Here, we demonstrated that targeting dCas9 to telomeres via sgTelo also induced a robust replication stress response at telomeres (Figures 2C–F), suggesting that the dCas9/sgRNA system is potentially the most versatile tool to induce replication stress at any genomic locus and both in vitro (28) and in vivo (this study). In addition, as demonstrated here, the dCas9/sgRNA system can also be used to investigate the downstream events of the replication stress response. For example, the formation of dicentric chromosomes, anaphase bridges, intercellular chromosome bridges, and potential genomic changes in the two daughter cells (steps VI and VII in Figure 1) and their progenies.

### Not all telomeres behave the same: chromosome arm-specific response to replication stress

One of the intriguing findings in this study is the differential responses to replication stress at telomeres among different chromosome arms and even between the two arms of the same chromosome (Figure 3, Supplementary Figures S5–S9 and Table 1). Given the 21-h induction time, it is not entirely surprising that at the population level, some of the characteristics studied exhibited no statistically significant differences between dCas9/sgNS and dCas9/sgTelo cells. On the other hand, we did notice the stark differences on individual chromosome arms for certain telomere/chromosome end features. For example, though there is no significant difference in the mean length of End Tel between the dCas9/sgNS cells and the dCas9/sgTelo cells, the telomeres of chromosome arms 18p and 20q reduce significantly in the dCas9/sgTelo cells compared to the dCas9/sgNS cells (Supplementary Figure S5). Conversely, the telomere of chromosome arm 14q is longer in the dCas9/sgTelo cells compared to the dCas9/sgNS cells (Supplementary Figure S5). One potential mechanism that regulates the chromosome arm-specific replication stress response may be differential expression of telomeric repeat-containing RNA (TERRA) or the formation of different amounts of TERRA R-loops. For example, several groups showed that different chromosome arms express different amounts of TERRA (47–53). We and others have demonstrated that aberrant accumulation of TERRA R-loops at telomeres can induce a robust replication stress response in ALT cells (54,55). Other possible reasons include the unique epigenetic and chromatin environments, the three-dimensional location within the nucleus, or the interaction/regulation of chromosomes with nuclear peripheral proteins. We do want to note that to define all the chromosome arm specific features with high confidence, including TFE, fusion/ITS– and fusion/ITS+, we will need to collect many more molecules in the future.

### Initiation of the BFB cycle by the replication fork stalling-/collapse-induced DSBs

One of the unanswered questions related to the BFB cycle is: how the initial DSB(s) in Step I of the BFB cycle is induced (Figure 1)? It has been well recognized that the DTR loci, including telomeres, are more prone to DNA damage than other regions of the genome (39–41). The primary cause of DNA damage at these DTR loci is likely due to the frequent replication fork pausing, stalling, or even collapsing, often due to the inherent sequences of the regions which can be prone to forming secondary structures. This in turn leads to the activation of the replication stress response and DNA damage. The data presented here indicates that telomeric replication stress-induced DSBs are likely the primary endogenous source of DSBs to initiate the first BFB cycle. Furthermore, we showed that cNHEJ, HDR and BIR promote the formation of ITCBs (Figure 7). BIR has previously been shown to be activated by a single one-ended DSB (56). Therefore, in theory, one unrepaired DSB from a single telomere has the latent potential to trigger BFB cycle with far reaching genomic consequences.

Finally, we demonstrated that inactivation of the TMEJ pathway led to an increase in the formation of ITCBs (Figure 7G). Consistent with our observation, two previous studies observed an increase in the frequency of inter-chromosomal translocation in Polθ deficient cells either induced by CRISPR/Cas9 (57) or at the endogenous Myc/IgH loci (58). In addition, Davis and colleagues demonstrated that Polθ deficient cells manifested an increase in the inter-homolog recombination (IHR), often leading to loss-of-heterozygosity (59). Collectively, these data suggest that TMEJ may play an essential role in suppressing tumorigenesis and tumor evolution by preventing the formation of dicentric chromosomes, thus the initiation of the BFB cycle through inhibiting IHR and inter-chromosomal translocation (step II in the Figure 1).

### Novel molecular and structural features of the intercellular chromosome bridges

Detection of an intercellular chromosome bridge in the intercellular canal during cytokinesis activates the abscission checkpoint to allow the cell more time to resolve the bridge (60). Because of the rarity of the intercellular chromosome bridge, its molecular and structural features remain largely unknown. Utilizing the dCas9/sgTelo-induced ITCBs, we have uncovered several novel features of the intercellular chromosome bridge. First, we discovered DNA knots in the middle of 86% of the ITCBs, which also colocalize with the midbody (Figure 5A). Most intriguingly, we demonstrated that approximately one third of the DNA knots are sites of active DNA repair and/or synthesis as indicated by EdU incorporation (Figure 5A-iv and Supplementary Figure S11). While this study was ongoing, the Zachos’ group reported their discovery of the DNA knots on either spontaneously generated or HU-induced intercellular chromosome bridges (61). Consistent with their findings, we also detected Top2A at the DNA knots (Figure 5A-v and Supplementary Figure S12), suggesting that over-twisted and/or catenated DNA is likely present in many ITCBs. In addition to Top2A, we also detected many other DDR proteins at different regions of the ITCBs, including the DNA knots (Figure 5 and Supplementary Figure S13). For example, the ssDNA binding protein, RPA, was detected at both the bodies and DNA knots of the ITCBs, suggesting that there are ssDNA gaps at both places. In addition to RPA, we also detected 53BP1, FANCI, and cGAS on the body of ITCBs. Interestingly, a previous study from Mitchison's group also detected cGAS on the body of the intercellular chromosome bridges (62). Their studies suggest that the intercellular chromosome bridge facilitates the activation of cGAS after drug-induced mitotic errors in human cells. The only protein that can be detected throughout the ITCBs is 53BP1. Its precise role in the formation and resolution of the ITCBs is currently under intense investigation.

In summary, the discoveries presented here have filled many knowledge gaps in the understanding of the BFB cycle. Because of the crucial role of the BFB cycle in the continued tumor evolution (4,5), which could lead to more significant tumor heterogeneity, treatment resistance, and relapse, our discoveries could also have significant implications in cancer treatments. For example, by suppressing the formation of ITCBs, cNHEJ, HDR and BIR inhibitors may slow down continued tumor evolution, reducing treatment resistance and tumor relapse. On the other hand, such intervention may risk further destabilizing the cancer genome because cNHEJ and HDR are so critical for DNA repair and genome maintenance. Many Polθ inhibitors are currently under development. Since Polθ inhibitor promotes the formation of ITCBs, it may accelerate tumor evolution, leading to increased treatment resistance and accelerated tumor relapse. Therefore, any therapy targeting DNA damage response and DNA repair will require careful consideration of the risk and benefit.

## Supplementary Material

gkae747_Supplemental_Files

## Data Availability

Raw and processed data reported in this paper will be available upon request.
